# Mitochondrial oxygen metabolism as a potential predictor of weight loss after laparoscopic sleeve gastrectomy for class III obesity

**DOI:** 10.3389/fendo.2024.1488175

**Published:** 2025-01-07

**Authors:** Markus Engelmann, Juliane Götze, Philipp Baumbach, Charles Neu, Utz Settmacher, Michael Ardelt, Hermann Kissler, Sina M. Coldewey

**Affiliations:** ^1^ Department of Anesthesiology and Intensive Care Medicine, Jena University Hospital, Friedrich-Schiller-University Jena, Jena, Germany; ^2^ Septomics Research Centre, Jena University Hospital, Friedrich-Schiller-University Jena, Jena, Germany; ^3^ Department of General, Visceral and Vascular Surgery, Jena University Hospital, Friedrich-Schiller-University Jena, Jena, Germany

**Keywords:** obesity, sleeve gastrectomy, bioimpedance analysis, mitochondrial dysfunction, mitochondrial oxygen metabolism, COMET

## Abstract

The prevalence of obesity is increasing at an alarming rate in industrialized countries. Obesity is a systemic disease that causes not only macroscopic alterations, but also mitochondrial dysfunction. Laparoscopic sleeve gastrectomy (LSG) poses a potential therapeutic option for patients with severe obesity. In order to ascertain the efficacy of bariatric interventions, it is important to assess not only weight loss, but also changes in body composition. Additionally, the aim of this study was to investigate the association between weight loss and cellular oxygen metabolism, a surrogate for mitochondrial function. We used bioimpedance analysis (BIA) to assess changes in weight and body composition in patients up to one year after LSG. To evaluate mitochondrial oxygen metabolism, we used the Cellular Oxygen Metabolism Monitor (COMET) to non-invasively measure the mitochondrial oxygen tension (mitoPO_2_), mitochondrial oxygen consumption (mitoVO_2_) and mitochondrial oxygen delivery (mitoDO_2_). We compared the values obtained in patients with obesity with those of age- and sex-matched healthy controls and investigated changes up to one year after LSG. 48 patients (46.5 years [35.5-55.3]; 38/48 female (79.2%); BMI 46.7 [42.5-51.0]) completed the study. They showed a significant weight loss and a decrease in relative fat mass after six months. We found no differences in mitochondrial oxygen metabolism between obese patients and healthy controls. MitoPO_2_, mitoVO_2_ and mitoDO_2_ did not change up to one year after surgery. It is noteworthy that patients who exhibited higher mitoPO_2_, mitoVO_2_, and mitoDO_2_ values prior to surgery demonstrated superior weight loss outcomes one year after LSG. This was the first study to investigate the non-invasively measured mitochondrial oxygen metabolism in the long-term course after bariatric surgery. Further studies in larger cohorts are needed to confirm these findings.

**Clinical trial registration:**

https://www.bfarm.de/DE/Das-BfArM/Aufgaben/Deutsches-Register-Klinischer-Studien/_node.html, identifier DRKS00015891.

## 1 Introduction

The World Health Organization defines obesity as abnormal or excessive fat accumulation with a body mass index (BMI) > 30 kg/m². Obesity is a growing health problem in most industrialized countries. The global economic impact of obesity and its associated complications, primarily due to an increased cardiovascular risk, was estimated at approximately US$ 1.96 trillion in 2020 and is projected to reach to US$ 4.32 trillion in 2035 (1). Obesity is associated with a range of significant health issues for patients. In addition to direct consequences such as reduced physical performance, there is an increased risk of developing secondary diseases associated with metabolic syndrome, including arterial hypertension, type 2 diabetes mellitus and dyslipidemia (2).

Once conservative treatment options, including lifestyle modifications, dietary counseling, and exercise have been exhausted, bariatric surgery may be considered as a potential intervention (3). A variety of surgical procedures may be employed to achieve weight loss, e.g. laparoscopic sleeve gastrectomy (LSG). LSG involves the excision of a portion of the stomach and the creation of a sleeve that has minimal capacity for food intake (3). A number of possible postoperative complications are provided by Sarkhosh et al. (4).

The primary objective of bariatric procedures is the achievement of weight loss. This outcome is most commonly quantified as either the loss of a proportion of total body weight (percentage total weight loss, %TWL) or excess weight (percentage excess weight loss, %EWL) (5, 6). A therapeutic success is considered as a %TWL ≥ 20 (7, 8) and a %EWL ≥ 50 (6, 9), respectively. Ideally, weight loss should only affect the excessive fat mass. The loss of fat-free mass, especially muscle tissue, is undesirable (10). Differentiated assessments of body composition can monitor this effect in post-operative follow-up assessments. An established technique is the bioimpedance analysis (BIA), a rapid, non-invasive method based on the measurement of the electrical impedance of a body by applying small, defined alternating electrical currents through electrodes placed on the patient’s extremities (11).

In addition to the macroscopic deleterious effects, there is evidence that obesity can also cause damage at the cellular level. Boudina et al. provide an overview of numerous studies assessing the association between mitochondrial dysfunction and obesity *in-vitro* and in mouse white adipose tissue (12). Prasun’s review goes on to describe the possible molecular mechanisms by which mitochondrial dysfunction may contribute to the development of metabolic syndrome, as well as potential mitochondrial-targeted therapeutic approaches (13).

It remains unclear whether mitochondrial dysfunction is a consequence or a cause of metabolic syndrome. Historically, the assessment of mitochondrial function was limited to invasive techniques i.e. the analysis of tissue biopsies. Mik et al. developed a non-invasive percutaneous method for measuring mitochondrial oxygen metabolism (14), which was subsequently developed into a clinical device, the Cellular Oxygen Metabolism Monitor (COMET). The method is based on the protoporphyrin IX-triplet state lifetime technique (PpIX-TSLT) and has provided reliable data on cellular oxygen metabolism as a surrogate for mitochondrial function in numerous studies in healthy volunteers and patients since it was first described in 2006 (15).

The objective of this study was to demonstrate the extent of weight loss and alterations in body composition resulting from LSG. Additionally, the study aimed to examine alterations in mitochondrial oxygen metabolism through non-invasive PpIX-TSLT measurements. This involved investigating whether there were differences between obese and non-obese individuals, whether these alterations changed postoperatively, and to what extent preoperative differences predicted the outcome of the surgery.

## 2 Materials and methods

This analysis is part of the prospective longitudinal single-center study “Body composition, mitochondrial oxygen metabolism and metabolome of patients with obesity before and after bariatric surgery (COMMITMENT)”. The study protocol has been published (16). The study was approved by the Ethics Committee of the Friedrich Schiller University Jena (Jena, Germany) on December 03, 2018 (2018-1192-BO).

### 2.1 Patient sample

Patients were recruited from February 2019 to February 2023 from the outpatient facilities of the Department of General, Visceral and Vascular Surgery of the Jena University Hospital, Jena, Germany. Adult patients with a BMI ≥ 40kg/m^2^, at least level 2 of the Edmonton Obesity Staging System with truncal obesity scheduled for LSG were included. Exclusion criteria were pregnancy and breastfeeding, participation in another interventional study, prior participation in this study, contraindications to COMET measurements including topical 5-ALA application such as porphyria, excessive photosensitivity and skin diseases aggravated by sunlight, and the presence of electronic implants as a contraindication to BIA measurements.

After giving informed consent, all patients underwent a preoperative assessment (T_0_), the LSG (T_1_) and four postoperative follow-up assessments: 3 ± 1 months (T_2_), 6 ± 1 months (T_3_), 9 ± 1 months (T_4_) and 12 ± 1 months (T_5_) after bariatric surgery.

The patient’s preoperative (T_0_) medical history and current medication were recorded. During the pre- and all postoperative visits, the patient’s current weight was recorded, fasting blood samples were taken and a BIA was performed. PpIX-TSLT measurements took place at T_0_, T_3_ and T_5_. In order to compare the PpIX-TSLT variables, we used a reference cohort of patients matched for age and sex from previous studies (17, 18).

### 2.2 Bioimpedance analysis

The BIA is a non-invasive measurement for estimating body composition. For the examination, the CE-certified Medical Body Composition Analyzer 525 (Seca GmbH & Co. KG, Hamburg, Germany) was used. We recorded the following variables: absolute fat mass (kg), relative fat mass (%), fat free mass (kg), skeletal muscle mass (kg), total body water (l), in addition the raw values of resistance (Ω), reactance (Ω) and the corresponding phase angle (°) were analyzed.

### 2.3 Non-invasive measurement of the mitochondrial oxygen metabolism

The CE-certified COMET system (Photonics, Healthcare BV, Utrecht, Netherlands) was used to non-invasively assess mitochondrial oxygen metabolism. The approach is based on the protoporphyrin IX-triplet state lifetime technique (PpIX-TSLT). The application of 5-aminolevulinic acid (5-ALA) substantially increases the concentration of PpIX, the final precursor of heme, in the mitochondria. After excitation by a light pulse, delayed fluorescence of PpIX can be observed. It is directly associated with the mitochondrial oxygen tension (mitoPO_2_) and is described by Stern-Volmer equation (19). The measurement of mitoPO_2_ has been calibrated and evaluated for different tissue types (e.g. heart, liver or skin) (20, 21) and is universally applicable (22).

MitoPO_2_, mitochondrial oxygen consumption (mitoVO_2_) and mitochondrial oxygen delivery (mitoDO_2_) were determined in a dynamic measurement according to a standardized measurement protocol: At least 4 hours before the measurement, a 5-ALA patch (Alacare^®^, approx. 4 cm², 8 mg, photonamic, Wedel, Germany) was applied to the clavipectoral triangle and covered with an opaque plaster. After measuring baseline mitoPO_2_ (mmHg), the sensor was pressed against the clavicle to determine mitoVO_2_ (mmHg/s). After oxygen in the tissue had been consumed (approx. 30 s), the pressure was released. MitoDO_2_ (mmHg/s) was calculated from re-oxygenation measurements (capillary re-filling, approx. 30 s). This procedure was repeated three times at each time point. The values of the PpIX-TSLT variables of the repeated measurements were averaged before the subsequent statistical analysis. The original measurement protocol allows the determination of the maximum and average mitoVO_2_ and mitoDO_2_ (17). We report average mitoVO_2_ and mitoDO_2_.

Data management, data preparation and estimation of PpIX-TSLT variables were performed using a self-developed script (Halley) in MATLAB (MATLAB and Statistics Toolbox Release 2017a, The MathWorks, Inc., Natick, Massachusetts, United States). A more detailed description can be found in (17).

### 2.4 Study endpoints

The primary endpoint of this study was the difference in relative fat mass between T_0_ and T_3_. The secondary endpoint was the difference in mitoVO_2_ between T_0_ and T_3_. We addressed the following additional endpoints:

- differences in variables of mitochondrial oxygen metabolism (mitoPO_2_, mitoVO_2_ and mitoDO_2_) between T_0_, T_3_ and T_5_
- explorative analysis of the association between BIA variables and variables of mitochondrial oxygen metabolism at T_0_, T_3_ and T_5_
- explorative cluster analysis: preoperative mitochondrial oxygen metabolism (T_0_) and prognostic relevance for variables of weight loss

### 2.5 Statistical analysis

For categorical variables, we report absolute (n) and relative frequencies (%). For metric variables, we report medians and interquartile ranges.

#### 2.5.1 Longitudinal comparisons

Differences in metric variables (BIA and PpIX-TSLT variables) between the study time points were tested using Wilcoxon-signed rank tests. The p values of the tests comparing multiple time points were adjusted using the Benjamini-Hochberg procedure [false-discovery rate adjustment (23)].

#### 2.5.2 Matching procedure and corresponding group differences

Healthy controls from the ICROS study (18) and PICOMET study (17) with available PpIX-TSLT measurements and BMI < 30 kg/m² were matched 1:1 for age and sex. We used the *optimal* clustering algorithm from the *MatchIt* R package. Comparisons between matched controls and patients on metric variables (BIA and PpIX-TSLT variables) were analyzed using Wilcoxon-signed rank tests.

#### 2.5.3 Explorative subgroup analysis and corresponding group differences

We used preoperative PpIX-TSLT variables (T_0_; mitoPO_2_, mitoVO_2_ and mitoDO_2_) as basis for *k*-medoids cluster analysis. The variables were z-standardized before clustering. The cluster analysis was performed with the *clusterboot* function of the fpc R package [Version 2.2-10; (24, 25)]. One hundred subsamples (each n = 2/3 of the original sample) were randomly selected from the total sample (n = 36). The number of possible clusters (*k*) was limited to 2-5 and the optimum for *k* was obtained using the Calinski-Harabasz index. Group differences in categorical variables were tested with Fisher’s exact tests. Differences in metric variables were analyzed using Mann-Whitney *U* tests.

For the analysis we used R [version 4.1.0, R Foundation for Statistical Computing, Vienna, Austria; (26)] and R Studio [version 1.4.1717, Boston, MA, USA; (27)]. Missing values were not imputed. In the case of missing values, the number of patients with non-missing data points is reported. We considered p values < 0.05 as statistically significant.

## 3 Results

### 3.1 Sample description

A total of 140 patients scheduled for LSG were screened between February 2019 and February 2023. Of these, 62 patients consented to participate in the study during the preoperative assessment. A total of 4 patients did not undergo surgery, and 7 patients withdrew from the study prior to the scheduled surgical procedure and associated T_0_ measurement.

**Figure 1:**
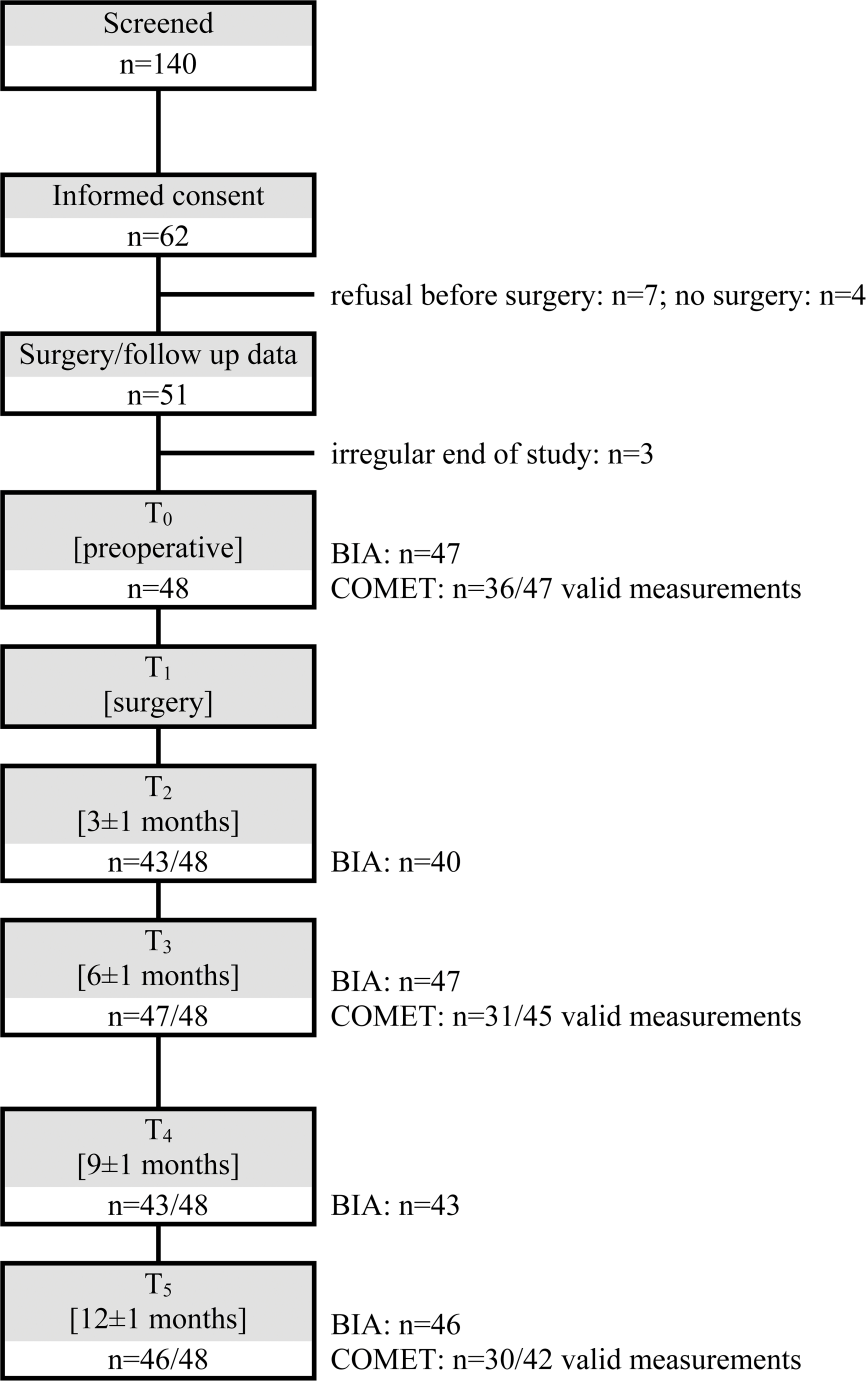
Flow chart. BIA, Bioimpedance analysis; COMET, Cellular Oxygen Metabolism Monitor for the assessment of the mitochondrial oxygen metabolism with protoporphyrin IX-triplet state lifetime technique.

Of the 51 patients who underwent T_0_, 48 patients completed the study in accordance with the study protocol. Two patients became pregnant during the study and one patient was lost to follow-up (**Figure 1**). The demographic data are presented in **Table 1**.

**Table 1:** Preoperative clinical data.

	Total sample N=48
Age (years)	46.5 [35.5–55.3]
Sex (female)	38 (79.2%)
Body height (cm)	168.0 [163.0–176.0]
Weight (kg)	128.5 [118.8–148.5]
Body Mass Index (kg/m²)	46.7 [42.5–51.0]
Excess weight (kg)	69.2 [57.2–80.0]
Years with obesity (years)	25.0 [21.0–37.0]; n=45
Diabetes mellitus	
no diabetes	35 (72.9%)
Type I	1 (2.1%)
Type II	12 (25.0%)
Oral medication	10 (20.8%)
Insulin therapy	6 (12.5%)
Arterial hypertension	36 (75.0%)
Medication	30 (62.5%)
Dyslipidemia	9 (18.8%)
Medication	3 (6.3%)
Number of Metabolic diseases^1^	
0	8 (16.7%)
1	24 (50.0%)
2	14 (29.2%)
3	2 (4.2%)
Obstructive sleep apnea (OSA)	
Yes	14 (29.2%)
Continuous positive airway pressure (CPAP)	14 (29.2%)
Gastroesophageal reflux disease (GERD)	15 (31.3%)
Medication	15 (31.3%)

All data in median [interquartile range] or absolute and relative frequencies (%).

^1^diabetes mellitus, arterial hypertension, dyslipidemia.

As depicted in **Figure 1**, measurement data were not available for all patients at every visit. This was due to pandemic-related restrictions of access to outpatient clinics, rare equipment malfunctions, and, predominantly in the case of PpIX-TSLT measurements, data sets that could not be evaluated due to poor measurement quality. To ensure a more reliable evaluation, we analyzed the time points T_3_ and T_5_ and also a trajectory sample comprising solely patients for whom results were accessible for all measurements.

### 3.2 LSG was associated with significant weight loss

As shown in **Table 2**, patients lost relevant weight after LSG. At T_3_, 37/47 (78.7%) patients exhibited %TWL ≥ 20% and 25/47 (53.2%) patients exhibited %EWL ≥ 50%. At T_5_ the numbers were further increased to 44/46 (95.7%) patients achieving %TWL ≥ 20% and 42/46 (91.3%) patients achieving %EWL ≥ 50%.

**Table 2:** Variables of weight loss.

	Visit	All patients	Trajectory N=33
Weight (kg)	T0	128.5 [118.8–148.5]; n=48	130.0 [120.0–148.0]
	T2	111.8 [104.8–126.5]; n=40	111.0 [104.0–126.0]
	T3	101.0 [89.5–110.1]; n=47	101.0 [90.0–109.6]
	T4	93.0 [82.0–104.5]; n=43	90.0 [82.0–102.9]
	T5	88.9 [80.1–99.1]; n=46	87.0 [80.1–98.0]
Body Mass Index (BMI, kg/m^2^)	T0	46.7 [42.5–51.0]; n=48	47.2 [43.6–51.7]
	T2	39.6 [36.6–44.1]; n=40	39.3 [35.9–43.1]
	T3	35.7 [32.0–38.8]; n=47	35.6 [32.0–38.8]
	T4	32.1 [29.5–36.0]; n=43	32.1 [29.5–35.2]
	T5	31.4 [28.9–33.6]; n=46	30.7 [28.5–33.4]
Excess weight (kg)	T0	69.2 [57.2–80.0]; n=48	70.0 [59.0–81.1]
	T2	50.6 [42.0–61.2]; n=40	49.9 [40.6–60.8]
	T3	39.2 [29.0–47.6]; n=47	38.0 [28.8–48.0]
	T4	29.7 [21.7–40.2]; n=43	29.7 [21.8–39.2]
	T5	26.1 [19.6–33.5]; n=46	26.0 [18.1–33.0]
Weight loss (kg)	T2	18.0 [16.0–24.3]; n=40	18.0 [15.9–25.0]
	T3	32.6 [25.4–35.7]; n=47	33.0 [25.9–35.3]
	T4	40.0 [31.8–45.1]; n=43	42.4 [34.4–47.0]
	T5	43.9 [32.8–50.0]; n=46	46.7 [40.0–52.4]
Total weight loss (%)	T2	14.4 [11.9–17.4]; n=40	14.8 [11.5–17.4]
	T3	23.7 [20.8–27.1]; n=47	24.0 [21.5–27.5]
	T4	29.5 [26.1–33.7]; n=43	31.0 [27.4–34.4]
	T5	33.8 [26.8–37.8]; n=46	36.5 [30.5–38.5]
Reduction in BMI (kg/m²)	T2	6.5 [5.5–8.7]; n=40	6.4 [5.6–8.8]
	T3	11.0 [9.3–12.8]; n=47	11.9 [9.6–13.1]
	T4	13.6 [11.2–16.5]; n=43	15.0 [12.9–16.7]
	T5	15.5 [11.5–17.8]; n=46	17.2 [14.6–18.4]
Excess weight loss (%)	T2	32.3 [26.3–37.0]; n=40	32.1 [26.4–36.8]
	T3	52.4 [44.5–59.7]; n=47	53.8 [46.3–60.2]
	T4	64.3 [55.6–73.7]; n=43	68.0 [57.7–75.4]
	T5	71.7 [61.7–80.6]; n=46	73.3 [65.6–83.8]

All data in median [interquartile range].

T0, preoperative; T1, day of surgery (no data acquisition); T2, 3 ± 1 months; T3, 6 ± 1 months; T4, 9 ± 1 months; T5, 12 ± 1 months after laparoscopic sleeve gastrectomy.

### 3.3 LSG was associated with significant loss of relative fat mass

**Figure 2:**
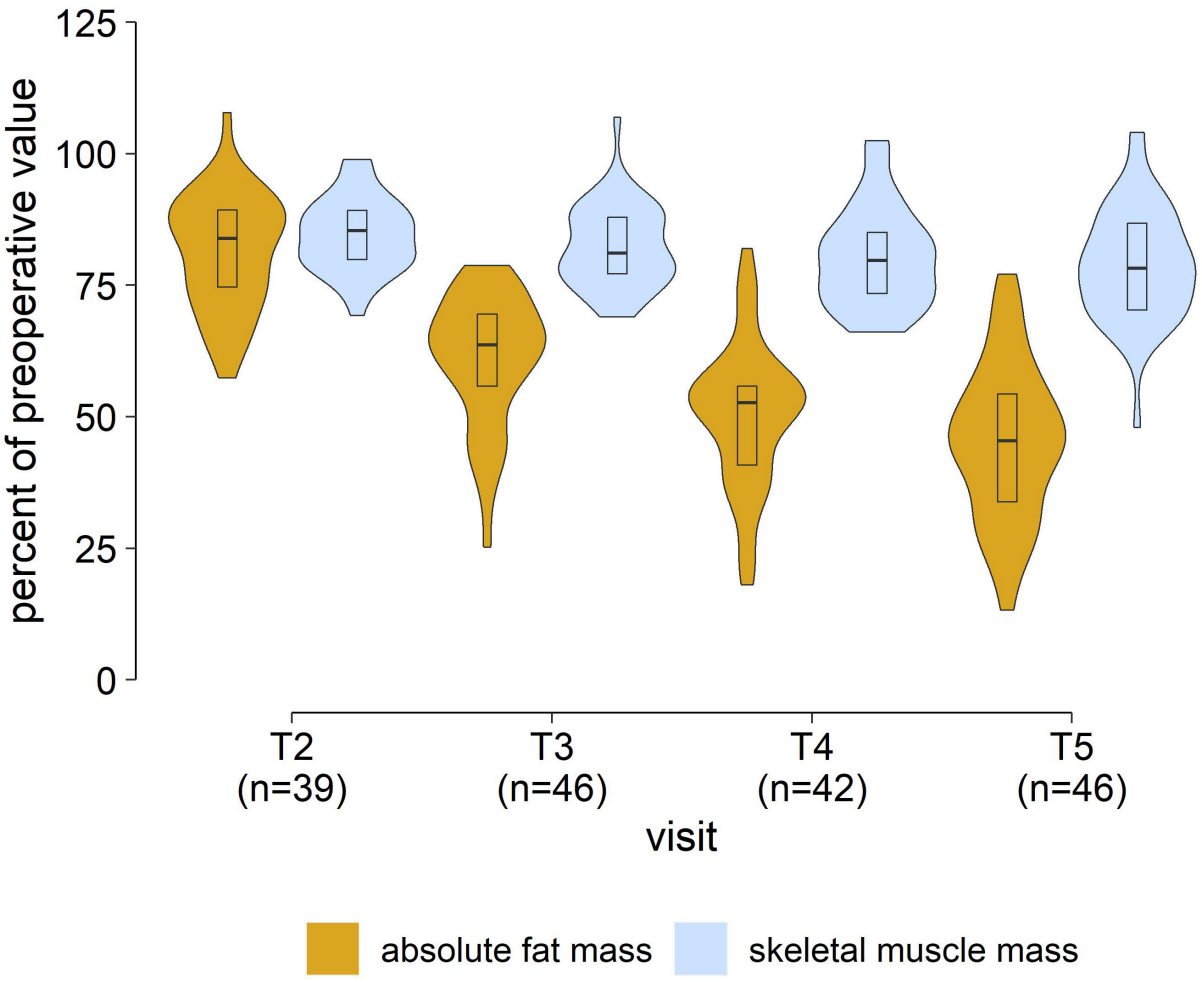
BIA measurements in patients. Changes in absolute fat mass and skeletal muscle mass in relation to preoperative values. [T_2_, 3 ± 1 months; T_3_, 6 ± 1 months; T_4_, 9 ± 1 months; T_5_, 12 ± 1 months after laparoscopic sleeve gastrectomy].

The relative fat mass decreased significantly between T_0_ (46.7% [41.1–50.2%]) and T_3_ (37.7% [32.6–44.4%]) n=46, p<0.001). Compared to the preoperative values, there was a decrease in fat mass over all time points while skeletal muscle mass was preserved (**Figure 2**). The results of the remaining BIA variables and time points are shown in **Table 3**.

**Table 3:** Results of the bioimpedance analyses.

	Visit	All patients	Trajectory N=33	FDR-adjusted p value				
				T0	T2	T3	T4	T5
Absolute fat mass (kg)	T0	58.2 [52.2−69.5]; n=47	63.8 [55.2−71.3]	–	*	*	*	*
	T2	50.9 [40.8−56.9]; n=40	52.8 [39.9−59.9]	*	–	*	*	*
	T3	37.6 [31.0−46.3]; n=47	40.2 [32.8−48.7]	*	*	–	*	*
	T4	32.6 [25.2−37.4]; n=43	32.6 [24.6−37.4]	*	*	*	–	*
	T5	26.6 [20.0−34.2]; n=46	27.1 [19.9−34.9]	*	*	*	*	–
Relative fat mass (%)	T0	46.5 [41.4−50.2]; n=47	48.5 [44.3−51.5]	–	ns	*	*	*
	T2	46.1 [38.5−50.1]; n=40	48.7 [38.7−51.0]	ns	–	*	*	*
	T3	38.2 [32.7−44.4]; n=47	40.8 [34.1−45.2]	*	*	–	*	*
	T4	34.4 [30.0−40.0]; n=43	36.2 [30.3−41.2]	*	*	*	–	*
	T5	32.3 [23.6−36.6]; n=46	34.4 [23.9−37.7]	*	*	*	*	–
Fat free mass (kg)	T0	70.8 [60.0−82.1]; n=47	67.2 [59.6−80.3]	–	*	*	*	*
	T2	62.5 [53.2−73.1]; n=40	58.2 [52.6−70.9]	*	–	ns	ns	ns
	T3	61.3 [53.4−72.6]; n=47	57.9 [50.9−67.7]	*	ns	–	ns	ns
	T4	58.9 [52.1−72.3]; n=43	58.9 [50.0−70.5]	*	ns	ns	–	ns
	T5	59.7 [52.4−71.7]; n=46	58.2 [51.3−65.0]	*	ns	ns	ns	–
Skeletal muscle mass (kg)	T0	35.2 [30.9−42.1]; n=47	33.7 [30.5−40.2]	–	*	*	*	*
	T2	30.8 [25.6−36.1]; n=40	28.5 [24.7−35.3]	*	–	*	*	*
	T3	28.9 [25.4−35.4]; n=47	27.7 [23.5−32.2]	*	*	–	ns	ns
	T4	27.3 [24.7−35.1]; n=43	27.1 [23.1−32.9]	*	*	ns	–	ns
	T5	27.3 [23.8−32.6]; n=46	26.6 [21.8−31.1]	*	*	ns	ns	–
Total body water (l)	T0	53.4 [45.7−61.7]; n=47	51.3 [45.5−60.4]	–	*	*	*	*
	T2	47.6 [40.6−54.4]; n=40	44.2 [39.6−53.1]	*	–	*	ns	ns
	T3	46.3 [40.5−54.2]; n=47	44.1 [38.7−51.3]	*	*	–	ns	ns
	T4	44.1 [39.1−54.0]; n=43	44.1 [37.6−53.4]	*	ns	ns	–	ns
	T5	44.7 [39.4−53.6]; n=46	43.8 [38.5−48.7]	*	ns	ns	ns	–
Phase angle (°)	T0	6.6 [6.1−7.4]; n=47	6.6 [6.0−7.4]	–	*	*	*	*
	T2	5.5 [5.0−6.3]; n=40	5.4 [5.0−6.4]	*	–	ns	ns	ns
	T3	5.4 [4.9−6.0]; n=47	5.3 [4.9−6.0]	*	ns	–	ns	ns
	T4	5.6 [5.1−6.2]; n=43	5.5 [5.0−6.2]	*	ns	ns	–	ns
	T5	5.7 [5.1−6.4]; n=46	5.6 [5.1−6.3]	*	ns	ns	ns	–
Resistance (Ohm)	T0	377.3 [333.5−412.5]; n=47	380.4 [347.3−413.0]	–	*	*	*	*
	T2	398.5 [347.7−456.8]; n=40	414.9 [359.8−458.9]	*	–	ns	ns	ns
	T3	394.3 [354.7−445.4]; n=47	423.5 [368.5−471.3]	*	ns	–	ns	ns
	T4	401.5 [356.7−444.7]; n=43	408.1 [364.9−458.1]	*	ns	ns	–	ns
	T5	409.5 [357.8−441.3]; n=45	416.4 [371.2−446.1]	*	ns	ns	ns	–
Reactance (Ohm)	T0	−44.7 [−48.5 to −39.0]; n=47	−44.7 [−48.2 to −40.2]	–	*	*	*	*
	T2	−39.9 [−43.9 to −36.1]; n=40	−40.5 [−44.1 to −37.6]	*	–	ns	ns	ns
	T3	−39.6 [−42.7 to −36.7]; n=47	−40.5 [−43.7 to −37.8]	*	ns	–	ns	ns
	T4	−40.3 [−44.5 to −35.4]; n=43	−40.3 [−45.1 to −35.7]	*	ns	ns	–	ns
	T5	−41.5 [−45.6 to −36.7]; n=45	−41.6 [−46.6 to −36.9]	*	ns	ns	ns	–

All data in median [interquartile range]. The last columns show significant differences between time points (Wilcoxon signed-rank tests, false discovery rate adjusted p values <0.05) in the trajectory sample with available data for all study visits.

T0, preoperative; T1, day of surgery (no data acquisition); T2, 3 ± 1 months; T3, 6 ± 1 months; T4, 9 ± 1 months; T5, 12 ± 1 months after laparoscopic sleeve gastrectomy.

*, false discovery rate adjusted p value <0.05; n.s., false discovery rate adjusted p value ≥ 0.05.

### 3.4 LSG was not associated with significant changes in mitochondrial oxygen metabolism

We found no significant differences in mitoVO_2_ between T_0_ (3.9 mmHg/s [2.4–5.2 mmHg/s]) and T_3_ (3.8 mmHg/s [2.7–4.6 mmHg/s], n=28 with paired data, p=0.45). There were no significant changes neither in mitoPO_2_, nor in mitoDO_2_ between T_0_ and T_3_. We also found no significant differences between T_0_ and T_5_. The results of the PpIX-TSLT variables and time points are shown in **Table 4**.

**Table 4:** Results of the PpIX-TSLT measurements.

	Visit	All patients	Trajectory N=21	FDR-adjusted p value		
				T0	T3	T5
mitoPO_2_ (mmHg)	T0	60.5 [47.8–78.0]; n=36	57.6 [50.0–68.8]	–	ns	ns
	T3	62.8 [47.9–76.1]; n=31	62.0 [47.9–73.2]	ns	–	ns
	T5	68.5 [58.8–91.3]; n=30	67.4 [57.8–91.7]	ns	ns	–
mitoVO_2_ (mmHg/s)	T0	3.8 [2.4–5.1]; n=36	3.6 [2.4–5.1]	–	ns	ns^†^
	T3	4.0 [2.9–4.7]; n=31	4.1 [2.6–4.7]	ns	–	ns
	T5	3.0 [2.3–4.4]; n=30	2.9 [2.1–3.8]	ns^†^	ns	–
mitoDO_2_ (mmHg/s)	T0	3.7 [2.4–5.9]; n=36	3.8 [2.7–6.3]	–	ns	ns
	T3	4.4 [3.2–6.9]; n=31	3.7 [3.0–7.1]	ns	–	ns
	T5	4.4 [3.2–6.3]; n=30	4.5 [3.4–6.0]	ns	ns	–

All data in median [interquartile range]. The last columns show significant differences between time points (Wilcoxon signed-rank tests, false discovery rate adjusted p values <0.05) in the trajectory sample with available data for all study visits.

mitoPO_2_, mitochondrial oxygen tension; mitoVO_2_, mitochondrial oxygen consumption; mitoDO_2_, mitochondrial oxygen delivery; PpIX-TSLT, Protoporphyrin IX-Triplet State Lifetime Technique; T0, preoperative; T3, 6 ± 1 months; T5, 12 ± 1 months after laparoscopic sleeve gastrectomy.

^†^ p < 0.05 before false discovery rate adjustment; n.s., false discovery rate adjusted p value ≥ 0.05.

#### 3.4.1 Feasibility and data quality

Patients applied the 5-ALA patch at least 4 hours prior to measurement. Despite adherence to the interval, the signal quality fluctuated, which in some cases resulted in uninterpretable measurements. For dynamic measurements (mitoVO_2_, mitoDO_2_), sufficient pressure against the clavicle was required to cut off the blood supply. This was not always feasible due to the presence of subcutaneous adipose tissue in this region, which can impede the accuracy of measurements. Consequently, only 76.6% of measurements were analyzable at T_0_, 68.9% at T_3_, and 71.4% at T_5_. This study presents a follow-up sample comprising patients who had analyzable measurements at all three time points.

#### 3.4.2 Comparison with control data

After matching, we found no significant differences between patients and controls regarding age (patients: 47 [37–56] years *vs* controls: 40 [29–64], p=0.87, both n=41) and sex (both groups n=33/41 females, 80.5%). The BMI differed significantly between patients (T_0_: 46.3 [42–50.8] kg/m²) and controls (23.4 [21.3–25.5] kg/m², p<0.001, both n=41).

**Figure 3:**
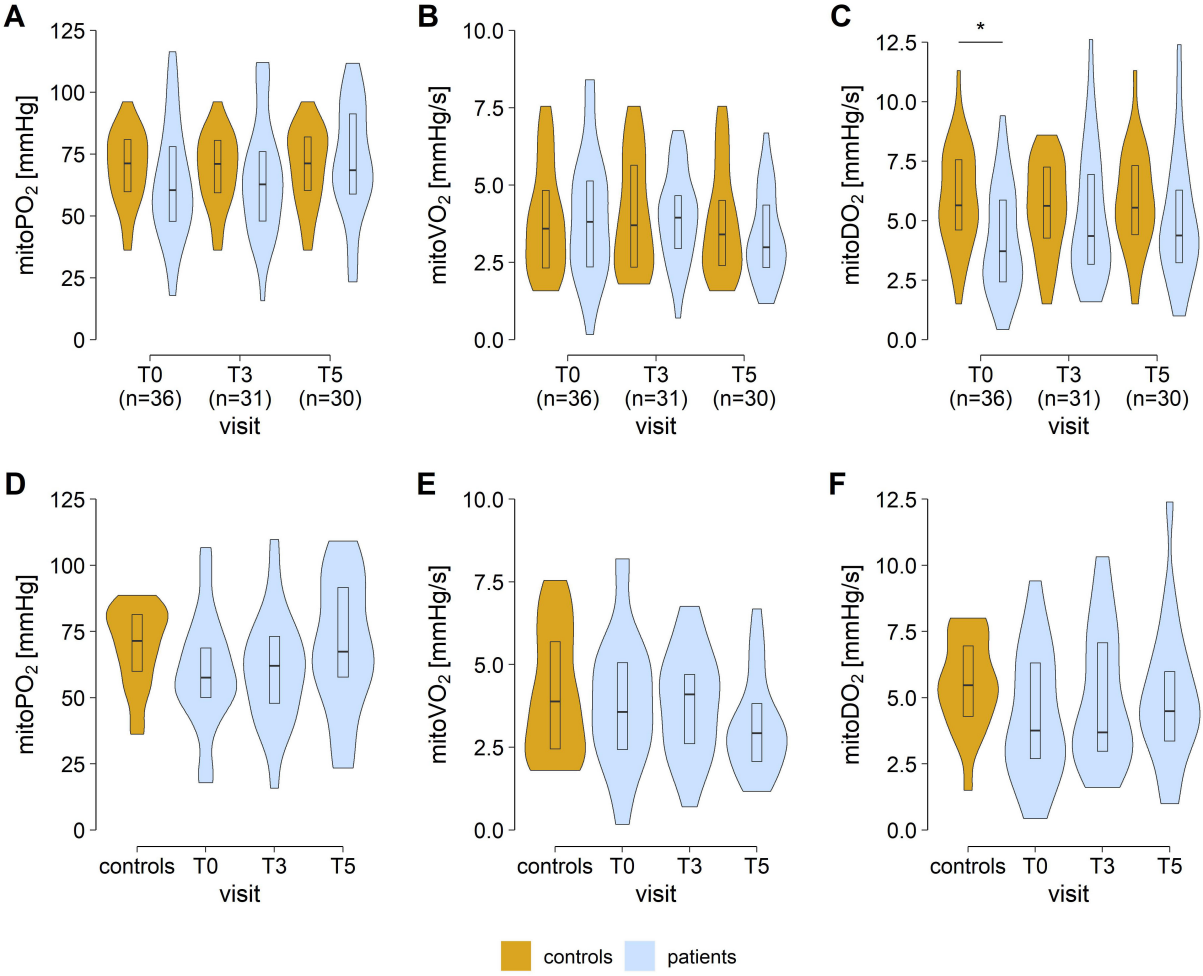
COMET measurements in patients and matched controls. Values of mitochondrial oxygen tension (mitoPO_2_, **A, D**), mitochondrial oxygen consumption (mitoVO_2_, **B, E**), and mitochondrial oxygen delivery (mitoDO_2_, **C, F**). Panels D to E show the results for the trajectory patient sample with available data for all time points (n=21). Asterisks mark false discovery rate adjusted p values <0.05 in group comparisons (Wilcoxon signed-rank tests). [T_0_, preoperative study visit; T_3_, 6 ± 1 months; T_5_, 12 ± 1 months after laparoscopic sleeve gastrectomy].

Apart from a significantly lower mitoDO_2_ at T_0_ (adjusted p value = 0.02), we did not find any significant differences between controls and patients with regard to PpIX-TSLT variables at any time point (all adjusted p values ≥ 0.05; **Table 5**; **Figure 3**).

**Table 5:** Comparison of PpIX-TSLT variables between patients and matched controls.

		All patients and matched controls	FDR adj. p value	Trajectory N=21	FDR-adj. P value
T0: preoperative					
Age (years)	controls	40.0 [29.0-64.0]; n=41	0.87	53.0 [31.0-65.0]	0.68
	patients	47.0 [37.0-56.0]; n=41		52.0 [37.0-57.0]	
Body Mass Index (kg/m²)	controls	23.4 [21.3-25.5]; n=41	**<0.001**	24.1 [21.5-26.0]	**<0.001**
	patients	46.3 [42.0-50.8]; n=41		43.6 [41.0-49.3]	
MitoPO_2_ (mmHg)	controls	71.3 [59.9-80.9]; n=36	0.25	70.1 [61.3-80.4]	0.29
	patients	60.5 [47.8-78.0]; n=36		57.6 [50.0-68.8]	
MitoVO_2_ (mmHg/s)	controls	3.6 [2.3-4.8]; n=36	0.86	3.6 [2.9-5.7]	0.97
	patients	3.8 [2.4-5.1]; n=36		3.6 [2.4-5.1]	
MitoDO_2_ (mmHg/s)	controls	5.7 [4.6-7.6]; n=36	**0.002**	5.4 [4.3-7.6]	0.29
	patients	3.7 [2.4-5.9]; n=36		3.8 [2.7-6.3]	
T3: 6 ± 1 months after surgery					
MitoPO_2_ (mmHg)	controls	71.0 [59.4-80.6]; n=31	0.64	70.1 [61.3-80.4]	0.29
	patients	62.8 [47.9-76.1]; n=31		62.0 [47.9-73.2]	
MitoVO_2_ (mmHg/s)	controls	3.7 [2.3-5.6]; n=31	0.86	3.6 [2.9-5.7]	0.74
	patients	4.0 [2.9-4.7]; n=31		4.1 [2.6-4.7]	
MitoDO_2_ (mmHg/s)	controls	5.6 [4.3-7.3]; n=31	0.64	5.4 [4.3-7.6]	0.68
	patients	4.4 [3.2-6.9]; n=31		3.7 [3.0-7.1]	
T5: 12 ± 1 months after surgery					
MitoPO_2_ (mmHg)	controls	71.3 [60.3-81.9]; n=30	0.86	70.1 [61.3-80.4]	0.78
	patients	68.5 [58.8-91.3]; n=30		67.4 [57.8-91.7]	
MitoVO_2_ (mmHg/s)	controls	3.4 [2.4-4.5]; n=30	0.64	3.6 [2.9-5.7]	0.29
	patients	3.0 [2.3-4.4]; n=30		2.9 [2.1-3.8]	
MitoDO_2_ (mmHg/s)	controls	5.6 [4.4-7.3]; n=30	0.43	5.4 [4.3-7.6]	0.68
	patients	4.4 [3.2-6.3]; n=30		4.5 [3.4-6.0]	

All data in median [interquartile range]. Results are presented for all patients with available data and the trajectory patient sample with available data for all study visits. False-discovery rate adjusted p values < 0.05 (Wilcoxon signed-rank tests) are printed in bold.

mitoPO_2_, mitochondrial oxygen tension; mitoVO_2_, mitochondrial oxygen consumption; mitoDO_2_, mitochondrial oxygen delivery; PpIX-TSLT, Protoporphyrin IX-Triplet State Lifetime Technique.

### 3.5 Association between PpIX-TSLT and BIA variables

At T_0_ and T_3_, no significant associations were identified between PpIX-TSLT and BIA variables (all raw p-values ≥ 0.05). At T_5_, higher values of mitoDO_2_ were associated with lower values of absolute (ρ=−0.39, p=0.034) and relative fat mass (ρ=−0.40, p=0.029). In view of the large number of correlations tested, these relationships should only be interpreted with caution (all adjusted p values ≥ 0.05; details in **Supplementary 01_ PpIX-TSLT_vs_BIA** in **Supplementary Table 1**).

### 3.6 Preoperative PpIX-TSLT variables were associated with long-term weight outcomes

#### 3.6.1 Correlative analyzes

At study visits T_2_, T_3_ and T_4_ no significant associations were identified between preoperative PpIX-TSLT variables (T_0_) and weight variables (including %EWL and %TWL). Higher preoperative values of mitoPO_2_ and mitoVO_2_ were associated with lower body weight, BMI, excess weight and higher excess weight loss at T_5._ However, after adjusting for multiple testing none of the p values was <0.05 (details in **Supplementary 02_ PpIX-TSLT_T0_vs_Outcome** in **Supplementary Table 1**).

#### 3.6.2 Subgroup analysis

**Figure 4:**
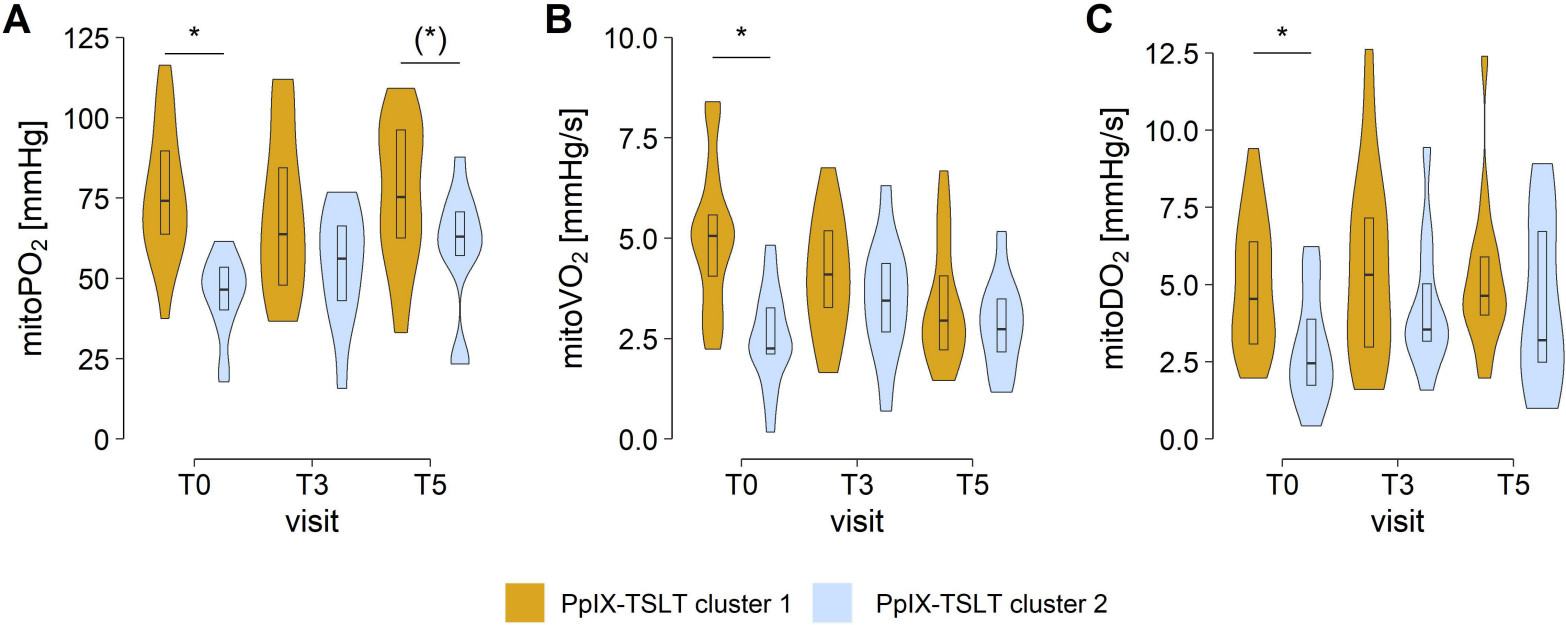
Preoperative clusters of COMET measurements. Group comparisons of **(A)** mitochondrial oxygen tension (mitoPO_2_) **(B)** mitochondrial oxygen consumption (mitoVO_2_), and **(C)** mitochondrial oxygen delivery (mitoDO_2_). Asterisks mark false discovery rate adjusted p values <0.05 in group comparisons. Asterisks in brackets indicate p values <0.05 before p value adjustment. [T_0_, preoperative study visit; T_3_, 6 ± 1 months; T_5_, 12 ± 1 months after laparoscopic sleeve gastrectomy]. PpIX-TSLT, Protoporphyrin IX-Triplet State Lifetime Technique.

In a cluster analysis of preoperative PpIX-TSLT variables two clusters were identified. Cluster 1 (n=21) was characterized by significantly higher preoperative levels of mitoPO_2_, mitoVO_2_ and mitoDO_2_ compared to cluster 2 (n=15, all FDR adjusted p values <0.05; **Table 6**; **Figure 4**). Following surgical intervention, no significant differences were identified in PpIX-TSLT variables between the two clusters. Additionally, apart from age, no significant differences were observed between the clusters with respect to demographic variables, preoperative weight-related variables and comorbidities.

**Table 6:** Characterization of the preoperative PpIX-TSLT clusters.

	Time	Cluster 1 N=21	Cluster 2 N=15	FDR adj. p value
Clinical variables				
Age (years)	T0	40.0 [34.0-52.0]; n=21	58.0 [51.5-61.0]; n=15	**0.045**
Sex (female)	T0	19 (90.5%); n=21	10 (66.7%); n=15	0.27
Body height (cm)	T0	166.0 [161.0-170.0]; n=21	171.0 [163.0-177.5]; n=15	0.46
Weight (kg)	T0	122.0 [113.0-130.0]; n=21	132.0 [123.5-149.0]; n=15	0.23
Body Mass Index (kg/m²)	T0	43.6 [41.3-47.1]; n=21	48.5 [44.0-50.6]; n=15	0.32
Excess weight (kg)	T0	59.4 [53.6-74.0]; n=21	75.9 [62.1-79.9]; n=15	0.27
Years with obesity (years)	T0	25.0 [16.5-34.0]; n=19	31.5 [22.0-40.0]; n=14	0.44
Diabetes mellitus	T0	3 (14.3%)	6 (40.0%)	0.27
Arterial hypertension	T0	13 (61.9%)	13 (86.7%)	0.27
Dyslipidemia	T0	3 (14.3%)	4 (26.7%)	0.46
OSA	T0	4 (19.0%)^a^	5 (33.3%)^a^	0.46
GERD	T0	7 (33.3%)^b^	6 (40.0%)^b^	0.74
PpIX-TSLT variables				
MitoPO_2:baseline_ (mmHg)	T0	74.2 [63.8-89.7]; n=21	46.6 [40.2-53.5]; n=15	**<0.001**
	T3	63.8 [48.0-84.5]; n=17	56.2 [43.1-66.4]; n=11	0.264
	T5	75.3 [62.6-96.3]; n=16	63.0 [57.2-70.7]; n=11	0.100**^†^**
MitoVO_2:avg_ (mmHg/s)	T0	5.1 [4.1-5.6]; n=21	2.3 [2.1-3.3]; n=15	**<0.001**
	T3	4.1 [3.3-5.2]; n=17	3.5 [2.7-4.4]; n=11	0.507
	T5	3.0 [2.2-4.1]; n=16	2.7 [2.2-3.5]; n=11	0.512
MitoDO_2:avg_ (mmHg/s)	T0	4.5 [3.1-6.4]; n=21	2.5 [1.7-3.9]; n=15	**0.010**
	T3	5.3 [3.0-7.2]; n=17	3.6 [3.2-5.0]; n=11	0.451
	T5	4.6 [4.0-5.9]; n=16	3.2 [2.5-6.7]; n=11	0.507
Weight variables				
Body weight (kg)	T2	105.5 [99.0-113.8]; n=17	113.0 [106.0-126.0]; n=13	0.36
	T3	92.0 [86.7-102.0]; n=20	103.0 [94.9-121.2]; n=15	0.12
	T4	83.1 [79.4-95.0]; n=20	102.4 [87.1-110.0]; n=13	0.07**^†^**
	T5	80.6 [76.4-86.6]; n=20	98.3 [88.4-103.6]; n=14	**0.028**
Body Mass Index (kg/m²)	T2	38.2 [34.4-42.8]; n=17	39.8 [38.2-43.1]; n=13	0.36
	T3	32.5 [30.7-37.6]; n=20	37.2 [32.6-39.1]; n=15	0.15
	T4	29.8 [28.8-34.1]; n=20	33.7 [31.8-36.3]; n=13	0.07**^†^**
	T5	28.2 [26.4-33.3]; n=20	32.8 [30.9-34.3]; n=14	**0.028**
Total weight loss (%)	T2	14.9 [12.5-18.0]; n=17	15.0 [12.4-18.0]; n=13	0.93
	T3	24.7 [21.4-28.1]; n=20	21.9 [18.8-25.6]; n=15	0.22
	T4	30.4 [27.4-34.4]; n=20	26.9 [24.1-29.6]; n=13	0.07**^†^**
	T5	35.6 [30.9-38.7]; n=20	28.1 [22.4-30.9]; n=14	**0.041**
Excess weight (kg)	T2	43.8 [34.2-56.1]; n=17	49.9 [43.9-60.8]; n=13	0.36
	T3	29.6 [25.8-42.3]; n=20	44.6 [30.4-49.9]; n=15	0.15
	T4	22.6 [18.8-33.7]; n=20	32.8 [27.0-40.7]; n=13	0.07**^†^**
	T5	17.2 [13.3-31.0]; n=20	32.1 [24.3-36.4]; n=14	**0.028**
Excess weight loss (%)	T2	32.8 [26.5-39.3]; n=17	33.9 [32.0-36.8]; n=13	0.85
	T3	54.7 [48.5-66.4]; n=20	48.4 [42.3-55.6]; n=15	0.15
	T4	71.2 [64.4-79.9]; n=20	57.7 [54.2-63.7]; n=13	**0.028**
	T5	84.1 [70.7-91.1]; n=20	62.2 [53.2-68.9]; n=14	**0.011**

All data in median [interquartile range] or absolute and relative frequencies (%). Differences in continuous variables were tested using Mann-Whitney *U* tests. For categorical variables, we applied Fisher’s exact tests. False-discovery rate (FDR) adjusted p values < 0.05 are printed in bold.

GERD, Gastroesophageal reflux disease; mitoPO_2_, mitochondrial oxygen tension; mitoVO_2_, mitochondrial oxygen consumption; mitoDO_2_, mitochondrial oxygen delivery; OSA, obstructive sleep apnea; PpIX-TSLT, Protoporphyrin IX-Triplet State Lifetime Technique; T0, preoperative; T1, day of surgery (no data acquisition); T2, 3 ± 1 months; T3, 6 ± 1 months; T4, 9 ± 1 months; T5, 12 ± 1 months after laparoscopic sleeve gastrectomy.

^†^ raw p value <0.05; p values were FDR adjusted separately for clinical, PpIX-TSLT, and weight variables.

**Figure 5:**
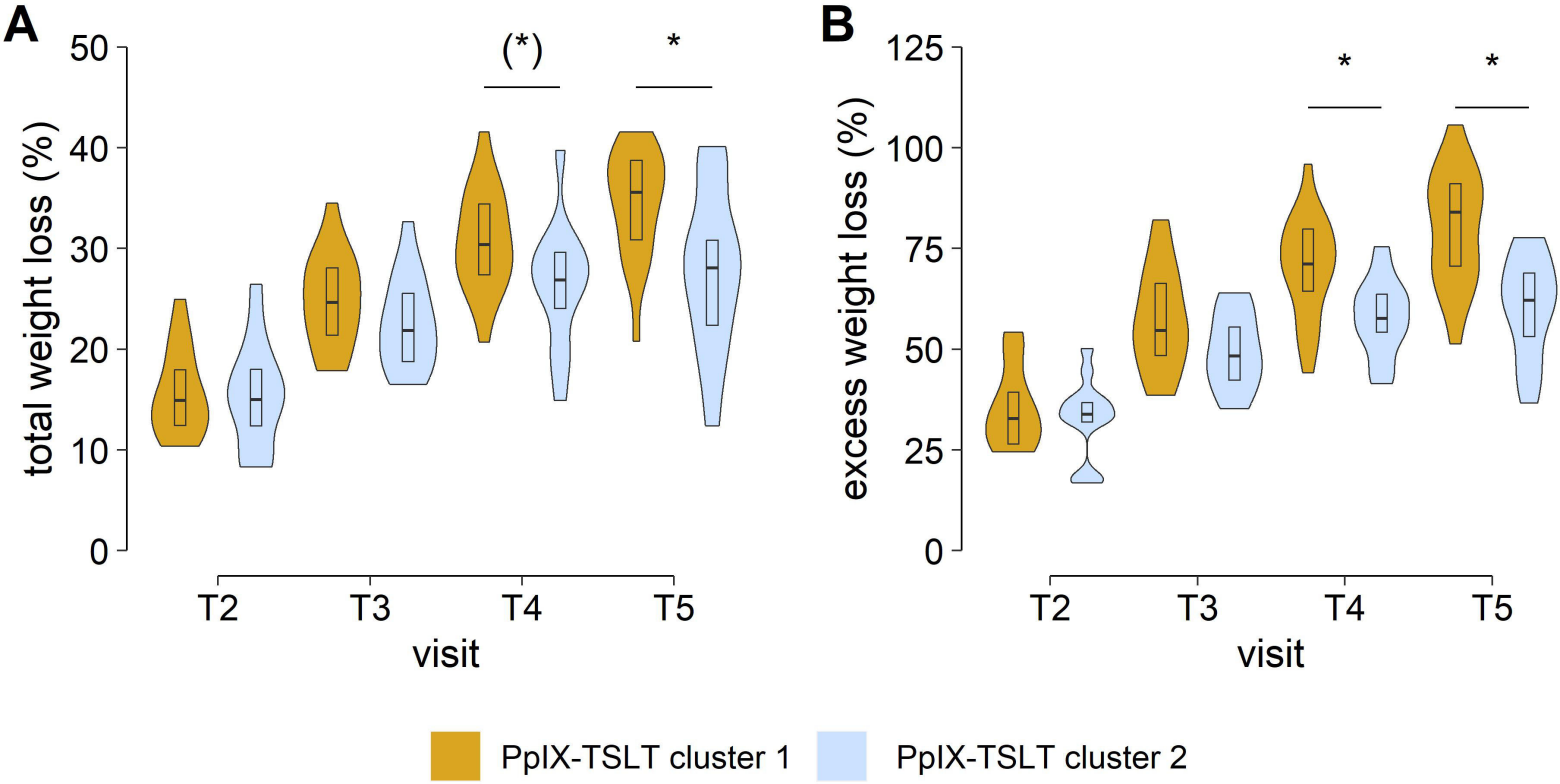
Clusters of COMET measurements. Longitudinal weight changes. Group comparisons of **(A)** total weight loss and **(B)** excess weight loss. Asterisks mark false discovery rate adjusted p values <0.05 in group comparisons. Asterisks in brackets indicate p values <0.05 before p value adjustment. [T_2_, 3 ± 1 months; T_3_, 6 ± 1 months; T_4_, 9 ± 1 months; T_5_, 12 ± 1 months after laparoscopic sleeve gastrectomy]. PpIX-TSLT, Protoporphyrin IX-Triplet State Lifetime Technique.

Patients in cluster 1 (*vs* cluster 2) showed better outcomes regarding weight and weight loss at T_4_ and in particular at T_5_ (**Table 6**; **Figure 5**). After adjusting for multiple testing, patients in cluster 1 (*vs* cluster 2) showed significantly lower body weight, BMI, and excess weight as well as higher total weight loss and excess weight loss one year after surgery (T_5_).

## 4 Discussion

The aim of this study was to determine the extent of weight loss and changes in body composition after LSG. In addition, changes in mitochondrial oxygen metabolism were to be examined by non-invasive PpIX-TSLT measurements. It was examined whether there are differences between obese and non-obese individuals, whether these alterations change postoperatively and to what extent preoperative differences predict the surgical outcome (16).

As anticipated, the patients exhibited a significant reduction in body weight six months after LSG, accompanied by a decline in relative fat mass as assessed through BIA. Mitochondrial oxygen metabolism demonstrated minimal alteration following surgery and did not diverge significantly from that observed in healthy controls. Interestingly, preoperative oxygen metabolism was a prognostic indicator for weight outcomes one year after surgery.

### 4.1 Bariatric surgery was associated with significant loss of weight and relative fat mass

LSG was found to result in a notable reduction in body weight among the patient cohort. Based on the benchmark variables %TWL and %EWL (6), the success rate was 90% in the studied cohort after 12 months and the majority of the weight loss could mainly be attributed to a reduction in fat mass. The observed weight loss following LSG in this study was consistent with the findings reported in the literature (28, 29). Concerning body composition, the cohort achieved a significant decrease in fat mass while preserving skeletal muscle mass. The magnitude of the loss in body weight, fat mass and fat-free mass was comparable to that reported in other studies (30, 31).

### 4.2 Results of the PpIX-TSLT measurements

#### 4.2.1 Obesity was only partially associated with alterations in mitochondrial oxygen metabolism

This work was the first to evaluate the mitochondrial oxygen metabolism during the long-term course after bariatric surgery. Based on the previous literature, we hypothesized that patients with obesity would show alterations in mitochondrial oxygen metabolism and that these alterations may change after LSG. To evaluate this hypothesis, we first compared the preoperative PpIX-TSLT variables in patients with class III obesity with an age- and sex-matched healthy control group from two previously conducted cohort studies (17, 18). A significant reduction in oxygen delivery (mitoDO_2_) was observed in patients with obesity prior to surgery compared to controls.

MitoDO_2_, initially described in healthy controls (17) and subsequently analyzed in patients with sepsis, has hitherto been regarded as a surrogate parameter of microcirculatory function. The observed reduction of mitoDO_2_ in patients with obesity is consistent with the finding of previous studies that have assessed the microcirculatory dysfunction in patients with obesity and demonstrated a prolonged recapillarization time in obese patients (32) and prolonged recovery time after supra-systolic occlusion measure by laser doppler in obese children (33). Nevertheless, this result should be viewed with caution, as it no longer occurs in the trajectory sample and the small sample size allows only limited conclusions.

With the exception of preoperative mitoDO_2_, we found no significant differences in PpIX-TSLT variables between matched controls and patients with obesity at any time point. Other studies using the PpIX-TSLT technique have primarily addressed the issue of mitochondrial damage in the context of macro- or microcirculatory disturbances typical of shock (15). The authors reported short-term differences in the PpIX-TSLT variables due to acute hemodynamic alterations. Mitochondrial function can be affected in the long term by metabolic changes such as those that occur in obesity (12, 13, 34–36), although the underlying pathophysiological mechanisms are not fully understood yet (13). It is possible that the mitochondrial changes in obesity are subject to different mechanisms than those in shock and therefore cannot be fully characterized by the determination of the PpIX-TSLT variables. The role of mitochondria in successful weight loss following bariatric surgery is currently under investigation (37).

#### 4.2.2 Bariatric surgery was only partially associated with significant changes in mitochondrial oxygen metabolism

A number of studies have demonstrated that mitochondrial dysfunction associated with obesity is potentially reversible (12, 13, 38). To investigate this phenomenon, we first compared PpIX-TSLT variables from patients who had undergone the LSG procedure six and 12 months previously with a control group of healthy individuals. Secondly, we analyzed the trajectory of PpIX-TSLT variables over time. Thirdly, we performed correlation analyses between weight-related variables and PpIX-TSLT variables. The preoperative reduction in oxygen delivery (mitoDO_2_) observed in patients was no longer detectable after six and 12 months, and was comparable to that of healthy controls. In line with the pre-operative results, the mitoPO_2_ and mitoVO_2_ values at six and 12 months after surgery remained comparable to those of the control group. Notwithstanding the substantial weight reduction, no notable discrepancies were observed in the PpIX-TSLT variables between the preoperative stage and the subsequent time points following LSG. Finally, in separate correlation analysis at T_0_, T_3_ and T_5_, most associations between PpIX-TSLT variables and weight-related variables were not statistically significant. However, at T_5_, higher mitoDO_2_ values were associated with lower fat mass. This result could be attributed to an improved microcirculation due to less subcutaneous adipose tissue. Given the absence of other reports on mitochondrial oxygen metabolism following bariatric surgery, no further conclusions can be drawn at this time, highlighting the necessity of additional studies in this area.

#### 4.2.3 Preoperative mitochondrial oxygen metabolism was a prognostic marker for long-term weight outcomes

In contrast to the aforementioned analyses, another objective was to ascertain whether the preoperative status of mitochondrial oxygen metabolism (PpIX-TSLT variables) is prognostically relevant for long-term weight-related outcomes following LSG. The findings of cluster analyses suggest that patients with higher preoperative values of mitoPO_2_, mitoVO_2_ and mitoDO_2_ (cluster 1) tend to exhibit more favorable weight-related outcomes compared to patients with lower preoperative PpIX-TSLT values (cluster 2). Age as a potential confounder (39) significantly differed between the clusters. However, in supplementary regression analysis controlling for age, baseline excess weight and diabetes status, patients in cluster 1 still showed more pronounced weight-related outcomes at T_5_ (**Supplementary 03_Regression_Analysis** in **Supplementary Table 1**). The higher weight loss may be attributed to higher mitochondrial oxygen metabolism. Larsen et al. showed an association of the oxygen affinity of isolated mitochondria with the basic metabolic rate in humans (40). Assuming that calorie intake is reduced equally due to the bariatric surgery, higher metabolic rate can lead to greater weight loss (41). However, at this time, we are unable to explain the biochemical mechanisms or pathways that underlie this relationship between mitochondrial oxygen metabolism and weight loss.

In general, the data on PpIX-TSLT variables, particular in the context of obesity, is still scarce. Our results indicate that further studies focusing on the predictive value of mitochondrial oxygen metabolism in obesity are warranted.

### 4.3 Limitations of the study

The monocentric design, limited sample size, and varying patient numbers, especially for PpIX-TSLT measurements, between the study visits limit the generalizability of the findings.

Despite the internal standardization of the PpIX-TSLT measurements, there was considerable variation in the quality of the data throughout the study. One source of error was the site of application of the 5-ALA patch. The infraclavicular fossa was selected for comparability with former studies conducted by our research group. However, due to subcutaneous adipose tissue, in some cases sufficient pressure could not be applied against the clavicle, making dynamic measurements (mitoVO_2_/mitoDO_2_) difficult. Therefore, the pre-sternal region should be selected for future measurements in obese patients. The patients were explicitly instructed to apply the patch the evening before the measurement and to document it accordingly. As demonstrated by Baysan et al., the measurement quality remains constant up to 24 hours after application. However, an exposure time of at least four hours must elapse before the measurement (42). This was maintained in all patients.

In accordance with the study protocol, follow-up visits were scheduled at intervals of three months. In order to ensure a high level of adherence to appointments, all study examinations were linked to follow-up visits in the bariatric surgery outpatient clinic. Unfortunately, many of these appointments were affected by pandemic-related access restrictions for outpatients, resulting in some visits not taking place within the targeted period or being canceled altogether.

In view of the limitations the results obtained should be regarded primarily as exploratory and hypothesis generating for future studies. Those should also investigate the extent to which PpIX-TSLT variables are associated with postoperative complications or interventions targeting mitochondrial metabolism (43).

## 5 Conclusion

The patients who underwent LSG demonstrated the expected weight loss 12 months post-operatively and exhibited a predominant loss of adipose tissue, while skeletal muscle mass remained unaltered. Variables of mitochondrial oxygen metabolism only partially differed significantly between patients and healthy controls preoperatively and did not significantly change after LSG. However, patients with higher values in variables of mitochondrial oxygen metabolism before LSG showed better weight-related long-term outcomes. Future studies should address this topic in larger patient cohorts and multicenter settings, in order to gain a deeper understanding of the mechanisms underlying the relationship between weight-loss and mitochondrial oxygen metabolism.

## Data Availability

The raw data supporting the conclusions of this article will be made available by the authors, without undue reservation.

## References

[1] Federation WO. World obesity atlas (2023). Available online at: https://www.worldobesityday.org/assets/downloads/World_Obesity_Atlas_2023_Report.pdf (accessed December 2024).

[2] Herrara MF Lozano-Salazar RR González-Barranco J Rull JA. Diseases and problems secondary to massive obesity. Eur J Gastroenterol Hepatology. (1999) 11:63–8. 10.1097/00042737-199902000-00002 10102212

[3] Colquitt JL Pickett K Loveman E Frampton GK. Surgery for weight loss in adults. Cochrane Database Syst Rev. (2014) 2014:Cd003641. 25105982 10.1002/14651858.CD003641.pub4 PMC9028049

[4] Sarkhosh K Birch DW Sharma A Karmali S. Complications associated with laparoscopic sleeve gastrectomy for morbid obesity: a surgeon’s guide. Can J Surg. (2013) 56:347–52. 10.1503/cjs.033511 PMC3788014 24067520

[5] Brethauer SA Kim J el Chaar M Papasavas P Eisenberg D Rogers A. Standardized outcomes reporting in metabolic and bariatric surgery. Surg Obes Relat Dis. (2015) 11:489–506. 26093765 10.1016/j.soard.2015.02.003

[6] van Rijswijk AS van Olst N Schats W van der Peet DL van de Laar AW. What is weight loss after bariatric surgery expressed in percentage total weight loss (%TWL)? A systematic review. Obes Surg. (2021) 31:3833–47. 10.1007/s11695-021-05394-x 34002289

[7] Corcelles R Boules M Froylich D Hag A Daigle CR Aminian A. Total weight loss as the outcome measure of choice after roux-en-Y gastric bypass. Obes Surgery. (2016) 26:1794–8. 10.1007/s11695-015-2022-y 26803753

[8] Grover BT Morell MC Kothari SN Borgert AJ Kallies KJ Baker MT. Defining weight loss after bariatric surgery: a call for standardization. Obes Surgery. (2019) 29:3493–9. 10.1007/s11695-019-04022-z 31256357

[9] Snyder B Nguyen A Scarbourough T Yu S Wilson E. Comparison of those who succeed in losing significant excessive weight after bariatric surgery and those who fail. Surg Endoscopy. (2009) 23:2302–6. 10.1007/s00464-008-0322-1 19184204

[10] Marks BL Rippe JM. The importance of fat free mass maintenance in weight loss programs. Sports Med. (1996) 22:273–81. 10.2165/00007256-199622050-00001 8923645

[11] Kyle UG Bosaeus I De Lorenzo AD Deurenberg P Elia M Gomez JM. Bioelectrical impedance analysis–part I: review of principles and methods. Clin Nutr. (2004) 23:1226–43. 10.1016/j.clnu.2004.06.004 15380917

[12] Boudina S Graham TE. Mitochondrial function/dysfunction in white adipose tissue. Exp Physiol. (2014) 99:1168–78. 10.1113/expphysiol.2014.081414 25128326

[13] Prasun P. Mitochondrial dysfunction in metabolic syndrome. Biochim Biophys Acta Mol Basis Dis. (2020) 1866:165838. 32428560 10.1016/j.bbadis.2020.165838

[14] Mik EG Stap J Sinaasappel M Beek JF Aten JA van Leeuwen TG. Mitochondrial PO2 measured by delayed fluorescence of endogenous protoporphyrin IX. Nat Methods. (2006) 3:939–45. 10.1038/nmeth940 17060918

[15] Mik EG Balestra GM Harms FA. Monitoring mitochondrial PO2: the next step. Curr Opin Crit Care. (2020) 26:289–95. 10.1097/MCC.0000000000000719 32348095

[16] Neu C Skitek K Kissler H Baumbach P Settmacher U Esper Treml R. Body composition, mitochondrial oxygen metabolism and metabolome of patients with obesity before and after bariatric surgery (COMMITMENT): protocol for a monocentric prospective cohort study. BMJ Open. (2022) 12:e062592. 10.1136/bmjopen-2022-062592 PMC9171273 35925679

[17] Baumbach P Neu C Derlien S Bauer M Nisser M Buder A. A pilot study of exercise-induced changes in mitochondrial oxygen metabolism measured by a cellular oxygen metabolism monitor (PICOMET). Biochim Biophys Acta Mol Basis Dis. (2019) 1865:749–58. 10.1016/j.bbadis.2018.12.003 30593898

[18] Coldewey SM Neu C Baumbach P Scherag A Goebel B Ludewig K. Identification of cardiovascular and molecular prognostic factors for the medium-term and long-term outcomes of sepsis (ICROS): protocol for a prospective monocentric cohort study. BMJ Open. (2020) 10:e036527. 10.1136/bmjopen-2019-036527 PMC7312455 32580988

[19] Ubbink R Bettink MAW Janse R Harms FA Johannes T Münker FM. A monitor for Cellular Oxygen METabolism (COMET): monitoring tissue oxygenation at the mitochondrial level. J Clin Monit Computing. (2017) 31:1143–50. 10.1007/s10877-016-9966-x PMC5655595 28000040

[20] Harms FA Bodmer SI Raat NJ Stolker RJ Mik EG. Validation of the protoporphyrin IX-triplet state lifetime technique for mitochondrial oxygen measurements in the skin. Opt Lett. (2012) 37:2625–7. 10.1364/OL.37.002625 22743475

[21] Mik EG Ince C Eerbeek O Heinen A Stap J Hooibrink B. Mitochondrial oxygen tension within the heart. J Mol Cell Cardiol. (2009) 46:943–51. 10.1016/j.yjmcc.2009.02.002 19232352

[22] Mik EG. Special article: measuring mitochondrial oxygen tension: from basic principles to application in humans. Anesth Analg. (2013) 117:834–46. 10.1213/ANE.0b013e31828f29da 23592604

[23] Benjamini Y Hochberg Y. Controlling the false discovery rate - a practical and powerful approach to multiple testing. J R Stat Soc B. (1995) 57:289–300.

[24] Hennig C. Cluster-wise assessment of cluster stability. Comput Stat Data An. (2007) 52:258–71.

[25] Hennig C. Dissolution point and isolation robustness: Robustness criteria for general cluster analysis methods. J Multivariate Anal. (2008) 99:1154–76.

[26] R Core Team. R: A language and environment for statistical computing. Vienna, Austria: R Foundation for Statistical Computing (2021).

[27] Team R. RStudio: integrated development for R. Boston, MA, USA: RStudio, PBC (2021).

[28] Palumbo P Banchelli F Miloro C Toschi PF Mecheri F Gabriele S. Weight loss trend after bariatric surgery in a population of obese patients. Clin Nutr ESPEN. (2023) 57:58–64. 37739709 10.1016/j.clnesp.2023.06.015

[29] Nuijten MAH Monpellier VM Eijsvogels TMH Janssen IMC Hazebroek EJ Hopman MTE. Rate and determinants of excessive fat-free mass loss after bariatric surgery. Obes Surg. (2020) 30:3119–26. 10.1007/s11695-020-04654-6 PMC7305251 32415634

[30] Dubnov-Raz G Inge TH Ben-Ami M Pienik R Vusiker I Yardeni D. Body composition changes in adolescents after laparoscopic sleeve gastrectomy. Surg Obes Relat Dis. (2016) 12:322–9. 10.1016/j.soard.2015.07.012 26525372

[31] Talalaj M Bogolowska-Stieblich A Wasowski M Binda A Jaworski P Wrzosek M. The influence of laparoscopic sleeve gastrectomy on body composition and fat distribution in obese caucasian men and women. Obes Surg. (2020) 30:3974–81. 10.1007/s11695-020-04766-z PMC7467906 32557384

[32] de Jongh RT Serne EH RG de Vries G Stehouwer CD. Impaired microvascular function in obesity: implications for obesity-associated microangiopathy, hypertension, and insulin resistance. Circulation. (2004) 109:2529–35. 10.1161/01.CIR.0000129772.26647.6F 15136505

[33] Schlager O Willfort-Ehringer A Hammer A Steiner S Fritsch M Giurgea A. Microvascular function is impaired in children with morbid obesity. Vasc Med. (2011) 16:97–102. 21393347 10.1177/1358863X11400780

[34] Bournat JC Brown CW. Mitochondrial dysfunction in obesity. Curr Opin Endocrinol Diabetes Obes. (2010) 17:446–52. 10.1097/MED.0b013e32833c3026 PMC5001554 20585248

[35] Kusminski CM Scherer PE. Mitochondrial dysfunction in white adipose tissue. Trends Endocrinol Metab. (2012) 23:435–43. 10.1016/j.tem.2012.06.004 PMC3430798 22784416

[36] de Mello AH Costa AB Engel JDG Rezin GT. Mitochondrial dysfunction in obesity. Life Sci. (2018) 192:26–32. 29155300 10.1016/j.lfs.2017.11.019

[37] Rossi MM Signorini FJ Castillo TA Parada MPS Moser F Baez MD. Sleeve gastrectomy reduces oxidative stress and reverses mitochondrial dysfunction associated with metabolic syndrome. Obes Surg. (2024) 34:2042–53. 10.1007/s11695-024-07244-y 38653888

[38] Lopez-Domenech S Abad-Jimenez Z Iannantuoni F de Maranon AM Rovira-Llopis S Morillas C. Moderate weight loss attenuates chronic endoplasmic reticulum stress and mitochondrial dysfunction in human obesity. Mol Metab. (2019) 19:24–33. 30385096 10.1016/j.molmet.2018.10.005 PMC6323177

[39] Contreras JE Santander C Court I Bravo J. Correlation between age and weight loss after bariatric surgery. Obes Surgery. (2013) 23:1286–9. 10.1007/s11695-013-0905-3 23462862

[40] Larsen FJ Schiffer TA Sahlin K Ekblom B Weitzberg E Lundberg JO. Mitochondrial oxygen affinity predicts basal metabolic rate in humans. FASEB J. (2011) 25:2843–52. 10.1096/fj.11-182139 21576503

[41] de Cleva R Mota FC Gadducci AV Cardia L D’Andrea Greve JM Santo MA. Resting metabolic rate and weight loss after bariatric surgery. Surg Obes Relat Dis. (2018) 14:803–7. 10.1016/j.soard.2018.02.026 29628405

[42] Baysan M Broere M Wille ME Bergsma JE Mik EG Juffermans NP. Description of mitochondrial oxygen tension and its variability in healthy volunteers. PloS One. (2024) 19:e0300602. 38829894 10.1371/journal.pone.0300602 PMC11146699

[43] Jiang S Yuan T Rosenberger FA Mourier A Dragano NRV Kremer LS. Inhibition of mammalian mtDNA transcription acts paradoxically to reverse diet-induced hepatosteatosis and obesity. Nat Metab. (2024) 6:1024–35. 10.1038/s42255-024-01038-3 PMC11199148 38689023

